# Problematic social Internet use and associations with ADHD symptoms in girls: a longitudinal observational study

**DOI:** 10.1186/s12889-024-20381-4

**Published:** 2024-10-16

**Authors:** Ashley Halkett, Stephen P. Hinshaw

**Affiliations:** 1 Department of Psychology, University of California, 2121 Berkeley Way West, Berkeley, CA 94704 USA; 2 Department of Psychiatry and Behavioral Sciences, UCSF Weill Institute for Neurosciences, University of California, San Francisco, CA 94143 USA

**Keywords:** Problematic internet use, Adolescent, Longitudinal, ADHD, Inattention, Social media

## Abstract

**Background:**

Problematic Internet use (i.e., Internet use that disrupts functioning in other important domains; PIU) is increasingly prevalent worldwide, particularly among youth. One form of PIU relates specifically to interpersonal interaction and communication, deemed *social* PIU. Social PIU has been linked to various forms of psychopathology, including attention-deficit/hyperactivity disorder (ADHD). Yet with limited longitudinal research, the direction of this association remains unclear. Moreover, little research investigates whether social PIU is linked to one or both symptom dimensions of ADHD (inattention vs. hyperactivity/impulsivity).

**Methods:**

The present study utilized data from the largest extant longitudinal study of girls with childhood-diagnosed ADHD (*N* = 228). Linear regression and structural equation models were used to analyze social PIU as both a prospective predictor and outcome of ADHD symptoms.

**Results:**

Inattentive ADHD symptoms were positively associated with concurrent social PIU in initial regression models but were non-significant in path analyses. Social PIU was only marginally significant in predicting subsequent inattention six years later. Symptoms of hyperactivity/impulsivity were unrelated to social PIU among girls at either time point.

**Conclusions:**

Inattentive ADHD symptoms were initially positively linked to concurrent problems with social Internet use, but bidirectional associations were non-significant in path analyses. Relations between PIU and ADHD in girls may be less robust than previously thought, although further longitudinal research with clinical samples is needed to clarify which groups of adolescents are particularly vulnerable to social PIU and its long-term effects.

## Introduction

It is difficult to overstate the ubiquity of the Internet in modern times. The International Telecommunication Union (ITU), a specialized agency dedicated to information technology, estimates that by the end of 2020, approximately 59% of the global population, or 4.6 billion people, were using the Internet [1]. Internet use is especially widespread among youth: globally, over 70% of youth and young adults aged 15–24 years were online by the end of 2020 [1]. It is entirely plausible that such usage rose even higher during the global COVID pandemic.

With the Internet fully integrated into the daily lives of young people, an emerging subgroup shows signs of problematic Internet use (PIU), reflecting the presence of, or potential for, negative repercussions stemming from Internet use. Although the overall amount of time spent online, or screen time, is typically assessed alongside PIU, most Internet researchers agree that PIU is not solely contingent on raw frequency of use but rather reflects a pattern of Internet-related behavior that disrupts the user’s functioning in other important domains [2]. The current study distinguishes between generalized PIU (related to any Internet-based activity) and social PIU, defined as PIU related specifically to social interaction and communication.

A major public health concern related to both generalized and social PIU among youth is associated psychopathology, often broadly defined. In the first longitudinal study on this topic, Kraut et al. (1998) investigated key outcomes associated with Internet use among families initially lacking Internet access. Greater social Internet use predicted increased loneliness and depression over time, adjusting for initial levels [3]. Beyond depression [4–7], generalized and social PIU have both been associated with anxiety disorders [8, 9], substance use disorders [10], and more recently, attention-deficit hyperactivity disorder (ADHD) [11–13].

ADHD is a prevalent neurodevelopmental condition characterized by symptoms of inattention, hyperactivity/impulsivity, or both [14]. Many ADHD-related symptoms, such as self-regulation difficulties (or disinhibition), a preference for immediate rewards, and feeling easily bored, have been shown to correlate with generalized PIU, supporting a link between the two [13, 15, 16]. Elevated rates of generalized PIU have been identified in samples of youth with ADHD (and vice versa), primarily in cross-sectional or case-control studies [8, 17, 18]. One early prospective study of Taiwanese adolescents found that ADHD was the most robust risk factor for new occurrences of PIU over two years [19]. Four subsequent longitudinal studies have identified ADHD as predictive of later PIU among adolescents [16, 20–22]. Most researchers argue that the symptoms of disinhibition and a preference for instant gratification, coupled with the widespread accessibility of the Internet, leave youth with ADHD especially susceptible to the development of generalized PIU [15].

Studies of ADHD and social PIU have emerged more recently, primarily focusing on problems with self-regulating social media use. Beyond the role of symptoms, ADHD may set the stage for social PIU by disrupting adolescents’ abilities to establish and maintain close relationships [23, 24]. Research suggests that youth with ADHD, particularly girls, face high rates of social rejection and exclusion [25, 26], potentially making social networking an attractive alternative to in-person interactions. Indeed, the evidence suggests a positive association between ADHD and adolescents’ problematic use of social media apps such as Facebook, Twitter, and Instagram [27–30]. Outside of social media apps, ADHD has also been linked to greater online interaction with both friends [12] and strangers [11]. However, a major limitation of this research is the overreliance on data from cross-sectional studies. In their systematic review of ADHD and digital media use, Thorell et al. (2022) explicitly call for more longitudinal studies focused on social Internet use, noting that they had identified only two longitudinal studies addressing social media specifically [31].

One unanswered question stemming from this gap is whether the presumed link between ADHD and social PIU is bidirectional. As noted above, some prospective research indicates that ADHD is a risk factor for later generalized PIU [16, 19, 20, 22]. However, in a three-wave longitudinal study, Boer (2020) found that youth whose ADHD symptoms increased over time did *not* show a parallel increase in problems with their social media use. Yet youth whose social media use problems increased *did* experience a corresponding increase in attention deficits at later time points—suggesting that problematic social media use may exhaust adolescents’ attempts at effortful control, ultimately weakening users’ abilities to maintain attention in offline settings [32]. This finding is also consistent with McNamee et al. (2021), who found that prolonged use of social media (> 4 h per day) predicted increased incidence of hyperactivity and inattention offline [29].

Another unanswered question is the extent to which the partially independent symptom dimensions of ADHD (inattention vs. hyperactivity/impulsivity) underlie the ADHD-PIU association. Some studies find that the inattentive symptoms of ADHD (IA) are predominantly associated with generalized PIU [17, 33, 34], yet other studies reveal a stronger link to hyperactive/impulsive symptoms (HI) [16, 20, 35]. A key issue is that much of the early literature does not address the actual activities in which users engage online. Given evidence for an especially robust link between ADHD and online gaming that could obfuscate findings on generalized PIU [36], additional research focusing on ADHD symptom dimensions in relation to more specific forms of PIU, including social PIU, is highly warranted. Such research would help to identify mechanisms linking ADHD and social PIU and could inform subsequent clinical efforts to reduce social media use in adolescents.

Clarifying potential mechanisms of influence is especially critical to promote healthy outcomes in adolescent girls, who are both more likely than boys to engage in social networking [37] and to suffer from poor mental health as a result [38]. Given heightened social difficulties among girls with ADHD, it is possible that ADHD serves to exacerbate the above issues by motivating girls to find alternative, non-traditional outlets for social interaction. One study of “multi-communicating,” or engaging in multiple virtual conversations at once, found that multi-communicating among girls (but not boys) with ADHD was strongly linked to a need for social assurance [12]. Girls with ADHD, therefore, may be uniquely vulnerable to developing social PIU as a result of both their symptomatology and their difficulty navigating friendships through typical (i.e., face-to-face) means. Untangling the associations between social PIU and ADHD symptom dimensions will allow for more targeted intervention efforts to reduce problematic use among this vulnerable population.

The current study examines social PIU and ADHD symptoms among a sample of adolescent girls with and without childhood diagnoses of ADHD. We hypothesized a bidirectional association between ADHD symptoms and social PIU, such that (a) concurrent ADHD symptoms will predict greater social PIU, and (b) greater social PIU will predict higher ADHD symptoms over time—approximately six years later. ADHD symptom dimensions (inattention and hyperactivity/impulsivity) were analyzed separately to elucidate potential differences in their relations to social PIU. Given the paucity of data on girls with confirmed histories of ADHD, we also present supplementary descriptive statistics on Internet-based social behaviors by childhood diagnostic group.

## Methods

### Participants and procedures

All data were drawn from the Berkeley Girls with ADHD Longitudinal Study (BGALS), an ongoing, prospective study of girls with childhood ADHD and an age- and ethnicity-matched neurotypical comparison group. Participants were recruited at age 6–12 years (*M*_*age*_ = 9.6 years) from schools, mental health care centers, pediatric practices, and direct advertisements to participate in research summer programs in 1997, 1998, and 1999. A total of 140 girls with ADHD and 88 neurotypical comparison girls were selected to participate, following extensive diagnostic assessments conducted via multiple informants and methods. Common psychiatric comorbidities (e.g., oppositional defiant disorder [ODD], conduct disorder [CD], anxiety disorders, depression, learning disorders) were allowed to promote the generalizability of the ADHD sample. The comparison group was also allowed to have internalizing disorders and ODD in order to prevent creating a supernormal comparison sample. All procedures were approved by the University’s Institutional Review Board. See Hinshaw (2002) for full details.

During the Wave 1 (W1) summer programs, multi-source data were collected from parents, teachers, behavioral observers, and program counselors on psychological, social, behavioral, cognitive, and familial functioning. The sample was racially and ethnically diverse (53% White, 27% African-American, 11% Latina, 9% Asian-American), and socioeconomic backgrounds ranged from professional parents to receipt of public assistance. Participants and their families were invited to engage in follow-up interviews that took place in three waves (W2, W3, W4), approximately 5, 10, and 16 years after baseline participation. Retention rates ranged from 92 to 95% at all follow-up points. Extensive statistical analyses reveal few significant differences between the retained and non-retained participants at each follow-up wave [24, 39, 40]. The current study utilizes data primarily from W3 and W4 to address the variables of interests, although several covariates were drawn from earlier waves (see Measures for details).

### Measures

#### ADHD diagnostic status & symptoms

W1 ADHD diagnostic status (present vs. absent) was determined from the Diagnostic Interview Schedule for Children (4th ed., DISC-IV) [41] and the Swanson, Nolan, and Pelham Rating Scale (4th ed., SNAP-IV) [42]. The DISC-IV is a well-validated, structured diagnostic interview administered to parents by highly trained research staff. The SNAP-IV is a well-validated, reliable measure frequently used as a screener for ADHD symptoms; SNAP-IV data were collected from parents and teachers prior to study enrollment. Participants were diagnosed as having ADHD if they met full diagnostic criteria for either ADHD-I or ADHD-C based on both the DISC-IV and parent SNAP-IV ratings.

At W1, ADHD symptom counts—separately for IA and HI—were obtained from parents using the DISC-IV and SNAP-IV. At all other timepoints (W2-W4), ADHD symptom counts were assessed on the SNAP-IV by both parent and participant report. For all measures, respondents were asked to rate ADHD symptoms during periods in which the participant was not taking ADHD medication.

#### Internet use (W3)

##### Social PIU

Use of the Internet for social interaction was measured using a 24-item self-report questionnaire found to be reliable and validated in previous research [11, 43]. Items in this questionnaire include statements directly endorsing a preference for socializing online as well as problems with self-regulating online social behavior. Preliminary factor analysis suggested a 3-factor solution, including one factor reflecting problematic use (7 items); these items were combined to form the subscale of problematic social Internet use (social PIU), the primary variable of interest. Sample items include “I have attempted to spend less time socializing online but have not been able to” and “I have routinely cut short on sleep to spend more time socializing online.” This subscale was found to have a Cronbach’s alpha of 0.87, 95% CI [0.84, 0.90], indicating good internal consistency. The two remaining factors reflected positive attitudes toward online communication (13 items) and a greater sense of control in online interactions (3 items). The Internet use measure also included 10 binary items assessing whether participants had ever engaged in specific Internet-based social behaviors, such as forming a romantic relationship with someone initially met online.

##### Screen time

Total screen time was measured at W3 with four questions assessing the average daily number of hours spent engaging in both synchronous and asynchronous social activities online (e.g., emailing friends, IM-ing with friends, posting on social network sites, updating personal blogs or webpages). Participants rated their daily hours for each of the four activities on a 5-point scale (0–1 h; 1–2 h; 2–3 h; 3–4 h; or 5 + hours).

#### Covariates

Path analyses included the following covariates: mother’s level of education on a 6-point scale (1 = less than 8th grade to 6 = advanced or professional degree), measured at W1; family yearly income on a 9-point scale (from 1 = less than $10,000 to 9 = $75,000 or more), measured at W1; participant age at W3; recent ADHD stimulant medication use (yes vs. no, between W2 and W3); and total screen time (average number of daily hours engaged in online activities), measured at W3. Demographic covariates were selected due to the diversity in age range and socioeconomic status in our sample, as well as research suggesting that Internet usage may vary significantly based on these factors [8, 33]. ADHD stimulant medication was included as a covariate given the unique sample characteristics. Total screen time was included as a covariate to provide a more conservative estimate of the specific effects of social PIU, rather than Internet use more broadly.

## Results

### Descriptive findings

Sample characteristics and data on Internet use by baseline diagnostic group are presented in Table 1. Girls with baseline ADHD diagnoses reported marginally significantly greater overall screen time (*p* = .050, d = 0.29) as well as more time spent on synchronous online socializing (e.g., IM-ing and emailing) (*p* = .022, d = 0.34). There was no diagnostic group difference with respect to social PIU.

**Table 1:** Full sample characteristics

Sample characteristics^1^	Full sample (*N* = 228)	Comparison (*N* = 88)	ADHD (*N* = 140)	*p* value	Effect size (Cohen’s d)
**Demographics**					
W1 age	9.6 (1.7)	9.4 (1.7)	9.6 (1.7)	0.377	
Race / ethnicity				0.040	0.36 [0.11, 0.62]
% White (not Latina)	52.6 (120)	46.6 (41)	56.5 (79)		
% Black	27.2 (62)	26.1 (23)	27.9 (39)		
% Latina	11.0 (25)	11.4 (10)	10.7 (15)		
% Asian-American	8.8 (20)	15.9 (14)	4.3 (6)		
% Native-American	0.4 (1)	0.0 (0)	0.7 (1)		
Income	6.4 (2.6)	6.7 (2.4)	6.2 (2.7)	0.105	
Maternal education	4.8 (1.0)	4.9 (1.0)	4.7 (1.0)	0.096	
% on stimulant since W2	33.5 (70)	1.2 (1)	54.0 (69)	0.000	1.15 [0.85, 1.44]
**Internet use**					
Social PIU	13.1 (4.4)	12.7 (4.0)	13.4 (4.7)	0.285	
Overall preference	20.2 (6.5)	18.0 (4.7)	21.8 (7.1)	0.000	0.64 [0.35, 0.94]
Desire for control	7.4 (2.5)	7.6 (2.5)	7.3 (2.5)	0.435	
Total screen time	6.7 (3.1)	6.2 (2.5)	7.1 (3.4)	0.050	0.29 [0.00, 0.57]
Time spent online socializing	3.3 (1.7)	3.0 (1.4)	3.6 (1.9)	0.022	0.34 [0.05, 0.62]

^1^ Data are presented as mean (SD) for continuous variables and percentage (N) for categorical variables of race and stimulant use

Additional descriptive data on specific Internet-based social behaviors are presented in Table 2. Girls in the comparison group were significantly more likely than girls with childhood ADHD to use the Internet at W3 to talk to same-aged friends whom they had initially met in person—both friends whom they saw often (*p* = .015, OR = 1.9; 95% CI = 1.1, 3.2) and not often (*p* = .016, OR = 1.9; 95% CI = 1.1, 3.2). In contrast, girls with childhood ADHD were significantly more likely to use the Internet at W3 to talk to people they met online and only knew online (*p* = .000, OR = 2.5; 95% CI = 1.5, 4.2), and to form close online relationships with people they had met online, both platonic (*p* = .011, OR = 2.0; 95% CI = 1.2, 3.3) and romantic (*p* = .000, OR = 2.4; 95% CI = 1.4, 4.1). Girls with childhood ADHD were also more likely to report having had an in-person romantic relationship with someone initially met online (*p* = .008, OR = 2.0; 95% CI = 1.2, 3.4) and using the Internet to talk to age-discrepant friends whom they don’t see often (*p* = .025, OR = 1.8; 95% CI = 1.1, 3.0).

**Table 2:** Internet-based social behaviors

Internet-based social activity (% endorsed, *N*)	Full sample (*N* = 228)	Comparison (*N* = 88)	ADHD (*N* = 140)	*p* value	Effect size (OR)
1. Ever talk to family members	59.3 (115)	55.0 (44)	62.3 (71)	0.386	
2. Ever talk to someone first met in person, same age, who you see often	80.0 (156)	88.9 (72)	73.7 (84)	0.015	1.90 [1.13, 3.19]
3. Ever talk to someone first met in person, same age, who don’t see often	74.4 (145)	84.0 (68)	67.5 (77)	0.016	1.89 [1.13, 3.17]
4. Ever talk to someone first met in person, different age, who you see often	54.4 (106)	53.1 (43)	55.3 (63)	0.877	
5. Ever talk to someone first met in person, different age, who you don’t see often	47.2 (92)	37.0 (30)	54.4 (62)	0.025	1.80 [1.08, 3.02]
6. Ever talk to someone first met online, who you only know online	34.5 (67)	20.0 (16)	44.7 (51)	0.001	2.49 [1.47, 4.21]
7. Ever formed a close online friendship with someone met online	26.2 (51)	16.0 (13)	33.3 (38)	0.011	1.95 [1.16, 3.28]
8. Ever formed an online romantic relationship with someone met online	12.3 (24)	2.50 (2)	19.3 (22)	0.001	2.41 [1.43, 4.08]
9. Ever formed an in-person romantic relationship with someone met online	16.4 (32)	7.40 (6)	22.8 (26)	0.008	2.02 [1.20, 3.40]
10. Ever had casual sexual activity with someone met online	8.7 (17)	4.90 (4)	11.4 (13)	0.187	

### ADHD predicting social PIU

First, we conducted generalized linear regression models to test whether concurrent (W3) ADHD symptom dimensions were significantly associated with social PIU at W3. With only these two symptom dimensions in the model, W3 IA symptoms were significantly positively associated with social PIU at W3 (β = 1.58, *p* = .015), but W3 HI symptoms were not. W3 IA symptoms remained significantly associated with social PIU after adjustment for both sociodemographic covariates and stimulant use. When screen time was added to the model, W3 IA symptoms remained a significant predictor of social PIU (β = 1.36, *p* = .043). Time spent online was also significantly positively associated with social PIU (β = 0.44, *p* = .000). This model explained 10.2% of the total variance and was statistically significant (*F* = 3.50, df = 148, *p* = .002).

We also adjusted the above model for baseline IA and HI symptoms as an additional test of robustness of W3 symptoms as predictor variables. With all variables included in the model, W3 IA symptoms remained significantly and positively associated with social PIU at W3 (β = 1.44, *p* = .037), as did total screen time (β = 0.44, *p* = .000). This final model explained 9.6% of the total variance and was statistically significant (F = 2.81, df = 145, *p* = .005).

### Social PIU predicting ADHD

Next, we used structural equation modeling to test for a bidirectional association between ADHD symptoms and social PIU, both with and without adjusting for covariates. Two path models were conducted, both including (1) W3 ADHD IA symptoms as a predictor of W3 social PIU, and (2) W3 social PIU as a predictor of W4 ADHD symptoms (both dimensions, measured approximately 6 years later). W4 ADHD symptom dimensions were analyzed as two separate outcomes to elucidate potential differences in the pathways between social PIU and each symptom dimension.

### Inattention

The first path model evaluated W3 ADHD IA as a predictor of W3 social PIU and W3 social PIU as a predictor of W4 ADHD IA symptoms, with and without adjusting for covariates. The model without covariates was a poor fit for the data. Adjustment for sociodemographic covariates and stimulant use resulted in improved model fit, but fit indices did not meet the threshold for acceptable model fit.

We then adjusted the second step of the path model to include W3 ADHD symptoms (both dimensions) as additional predictors of W4 ADHD symptoms. This allowed for our model to estimate a direct path from ADHD symptoms at W3 to W4. Adjustment for W3 ADHD symptoms resulted in a fully saturated model with perfect fit (CFI = 1.000, TLI = 1.116, RMSEA = 0.000, CI_90_=[0.000, 0.111], SRMR = 0.004).

Examining the coefficients of our variables of interest, W3 social PIU was not a significant predictor of W4 ADHD IA symptoms (β = 0.02, *p* = .055), although the trend was in the expected direction. The association between W3 ADHD IA symptoms and W3 social PIU also dropped to marginal significance, β = 1.24, *p* = .066. Unsurprisingly, the pathway from W3 ADHD IA symptoms to W4 ADHD IA symptoms was highly significant, β = 0.50, *p* = .000. Additional covariates reaching statistical significance as predictors of W4 ADHD IA symptoms were W1 income (β =-0.04, *p* = .020), age at W3 (β = 0.05, *p* = .038), and screen time (β =-0.04, *p* = .001).

### Hyperactivity/Impulsivity

We repeated the steps outlined above, but with W3 social PIU as a predictor of W4 ADHD HI symptoms. As with our first model predicting W4 ADHD IA symptoms, this model without covariates was a poor fit for the data. Adjustment for sociodemographic covariates and stimulant use similarly resulted in improved model fit, but did not meet the threshold for acceptable model fit. Adjustment for W3 ADHD symptoms, however, resulted in a fully saturated model with perfect fit (CFI = 1.000, TLI = 1.007, RMSEA = 0.000, CI_90_=[0.000, 0.159], SRMR = 0.006).

Examining the coefficients of our variables of interest, W3 social PIU was not a significant predictor of W4 ADHD HI symptoms, β = 0.01, *p* = .512. The pathway from W3 ADHD HI symptoms to W4 ADHD HI symptoms was significant, β = 0.49, *p* = .000, as was the pathway from W3 ADHD IA symptoms to W4 ADHD HI symptoms, β = 0.14, *p* = .044. No covariates were statistically significant predictors of W4 ADHD HI symptoms.

## Discussion

We examined ADHD symptom dimensions as both predictors and outcomes of social PIU in adolescent girls. ADHD IA symptoms, but not HI symptoms, were positively associated with concurrent social PIU in regression analyses. However, structural equation modeling revealed that when the direct pathway from W3 ADHD IA symptoms to W4 ADHD IA symptoms was considered, W3 social PIU did not significantly predict W4 ADHD IA symptoms (*p* = .055). The initial association between W3 ADHD IA symptoms and W3 social PIU also dropped to marginal significance in path analyses. Finally, W3 social PIU was unrelated to ADHD symptoms of hyperactivity/impulsivity at either time point.

These findings suggest that the link between social PIU and inattention may be less robust than previously thought, especially when accounting for related factors. That said, our results are generally consistent with previous findings indicating that deficits in attention are more likely to play a role in Internet addiction among youth than are problems with hyperactivity/impulsivity [17, 34]. Indeed, unlike inattention, ADHD HI symptoms in our participants were unrelated to social PIU in either regression or path analyses. One possible explanation is that ADHD HI symptoms contribute to generalized, but not social, PIU. Measures of generalized PIU encompass a broad range of online activities, including gaming, shopping, and gambling, all of which have been linked to clinically significant levels of impulsivity [36, 44]. It may be that ADHD HI symptoms contribute to difficulties self-regulating certain online activities while playing a more limited role in social PIU. It is also possible that hyperactivity/impulsivity is less relevant in understanding social PIU among adolescent girls compared to boys, who are overrepresented in studies of ADHD and generalized PIU. Indeed, one study of computer gaming disorder found that when participants were stratified by gender, inattention was the second most robust predictor of gaming disorder for girls versus hyperactivity/impulsivity for boys [45]. In the current all-female sample, HI symptoms could have played a less important role than in studies with all-male or mixed-gender samples—especially regarding social PIU, as boys on average report higher levels of all online activities *except* for social networking [37].

The current findings are somewhat at odds with a growing body of research indicating that both generalized and social PIU could contribute to later problems with attention. We found that social PIU was only marginally significant in predicting ADHD IA symptoms several years later. These results contrast with one of the few other longitudinal studies in this area, which found that Dutch youth whose problems with social media use increased from T1 to T2 experienced a subsequent increase in attention deficits from T2 to T3 [32]. Another longitudinal study of Japanese adolescents found a bidirectional association between generalized PIU and a combined index of hyperactivity/inattention [21]. One possible explanation for these discrepant findings is that both prior studies were conducted with community-based populations and relied solely on self-report questionnaires to assess ADHD symptoms, whereas our sample included girls with clinical diagnoses of ADHD. It is possible that social PIU is linked to inattention in general youth populations but does not contribute to symptom exacerbation among adolescents who already show clinically elevated levels of ADHD. Future research with clinical samples will be necessary to determine whether the impact of social PIU on inattention is moderated by diagnostic history, which would be highly relevant for intervention efforts.

Regarding more specific Internet-based social behaviors, neurotypical girls and girls with ADHD also diverged significantly regarding with whom they socialized online. Girls in the comparison group were more likely to use the Internet to socialize with people they had initially met in-person, whereas girls with ADHD were more likely to initiate and maintain new relationships entirely online. This finding is consistent with previous research that youth with ADHD are more likely to use Facebook to meet new people and to initiate (and terminate) romantic relationships [46]. Such data suggest that, even if ADHD symptoms are not significantly associated with a global measure of social PIU, adolescents’ actual behaviors may manifest differently depending on their histories of childhood ADHD.

Several methodological considerations should be highlighted as both limitations and strengths of the current study. First, with respect to limitations, the current sample of adolescent girls living in the Northern California Bay Area is not representative of the general population of youth, or even youth with ADHD. Considering that girls appear more likely both to engage in social Internet use [27, 32] and to suffer poor mental health as a result [29, 47], a special focus on this population is warranted. However, as noted previously, the findings reported herein may be of less applicability to adolescent boys, given some evidence for sex differences in the etiology of generalized PIU [6].

Another limitation is that these data on social Internet use were collected between 2007 and 2010, several years after Facebook was launched but prior to the current ubiquity of social media apps. Thus, the current results cannot speak to the correlates or consequences of social media as it currently exists. Smartphones, moreover, were not nearly as prevalent during the time of data collection as they are now. Indeed, the Pew Research Center only began collecting statistics on smartphone ownership in 2011, when just 35% of respondents owned a smartphone [48]. Although we did not ask our participants how they accessed the Internet, it is reasonable to assume that between 2007 and 2010, the vast majority were logging online via computers, which are inherently less accessible than mobile smartphones. Indeed, given that modern smartphones provide instantaneous access to social media apps and often interrupt users’ attention with incoming notifications, it is quite possible that the current findings underestimate the true strength of associations between social PIU and inattentive symptoms [49].

A key strength of the current study is the utilization of longitudinal, prospective data, which are unfortunately rare in studies of Internet use and psychopathology [50]. These data allowed for a more nuanced investigation of temporal associations between ADHD symptoms and social PIU, as well as adjustment for baseline symptomatology and sociodemographic factors previously found to influence social media use [29]. In addition, ADHD in the current sample was carefully, rigorously diagnosed by clinical experts, with symptoms assessed using multiple informants, minimizing the possibility of inaccurate self-reports of ADHD. Analyses also adjusted for participants’ use of stimulant medication, yielding more conservative estimates of the effects of current ADHD symptoms on social PIU. Finally, the inclusion of total screen time as a covariate allowed for greater isolation of the specific prospective link from social PIU to later ADHD symptoms—especially important as PIU is often conflated with the raw amount of time spent online, despite evidence that ADHD is linked to problematic rather than purely excessive use [31, 32].

## Conclusions

The current results suggest that long-term associations between social PIU and inattention may be less robust than previously thought. Although ADHD IA symptoms and social PIU were significantly correlated in regression analyses, even after adjustment for covariates, this link was not significant in path analyses assessing bidirectionality. Furthermore, social PIU was only a marginally significant predictor of later inattention in our all-female sample. Much of the existing research on Internet use and mental health examines anxiety and depression as outcomes of social PIU, with far less attention paid to cognitive functioning. Longitudinal studies with both clinical and community samples would help to clarify the types of Internet users most vulnerable to subsequent mental health issues and facilitate the development of targeted interventions for youth with social PIU.

## Data Availability

No datasets were generated or analysed during the current study.
